# Supplementary material for: The Hospital Anxiety and Depression Scale (HADS) applied to Ethiopian cancer patients
Source: PLoS One. 2020 Dec 3;15(12):e0243357. doi: 10.1371/journal.pone.0243357 (PMC7714130; doi:10.1371/journal.pone.0243357)
Supplement: Attachment — Submitted filename: Responses to Reviewers.docx [file pone.0243357.s002.docx]

**Responses to Reviewers**

We thank the reviewers very much for their valuable comments.

**Editor:**

Please address the issues raised regarding disparate sample sizes and how the problem was addressed in the analyses

**Response**. See response to reviewer #2 on page 4.

Please upload a copy of Figure 2, to which you refer in your text on page 13. If the figure is no longer to be included as part of the submission please remove all reference to it within the text.

**Response**. It was a mistake, we should have written Figure 1. Thank you for this remark, we corrected it.

**Reviewer #1. PLOS Review (Attachment):**

**The Hospital Anxiety and Depression Scale (HADS) applied to Ethiopian cancer patients**

The study is very interesting in describing the HADS-results in Ethiopian cancer patients.

Unfortunately, there are many aspects which should be considered.

**32 the present study was to test the Hospital Anxiety and Depression Scale (HADS) in a sample**

**33 of Ethiopian cancer patients and to compare the results with those obtained from a**

**34 comparable sample in Germany.**

It is not clear whether the sample from Germany really is “comparable”

**Response**. The German sample is comparable concerning the age and gender distribution, but concerning other criteria there are differences. Therefore, we removed the word “comparable”.

**40 (depression), and 0.91 (total scale). The Ethiopian cancer patients were more anxious (M =**

**41 7.9) and more depressed (M = 9.3) than the German patients (M = 6.8 for anxiety and M = 5.5**

**42 for depression).**

This conclusion is depending on a correct matching (see below)

**Response**. There are several options for matching. The more criteria included, the lower the size of the remaining comparison group. We believe that age and gender cover two relevant dimensions, omitting the problem that old German cancer patients were compared with younger Ethiopian patients, and that a sample with 61 % females (Ethiopia) is compared with a sample with a nearly balanced gender distribution (Germany). Of course, differences remain, concerning other sociodemographic (education urban/rural) and clinical (tumor type, stage, treatment) variables. It is not possible to consider all of these variables. Moreover: If we had reduced the number of well-educated people and people living in urban areas in the German cancer population (to make it better comparable with the Ethiopian distributions), we would arrive at a rather untypical sample of German cancer patients. Therefore, we preferred to use only the two criteria, age and gender, for matching.

In the Limitations section we mentioned the issue of the imperfect match between the samples.

**43 Ethiopian and the German sample. In the Ethiopian sample, anxiety and depression were**

**44 associated with tumor stage (high levels in stage 4) and treatment (high levels for patients not**

**45 receiving surgery and chemotherapy).**

What are the corresponding results in the German patients ?

What are the general distribution of cancer types, stages and other variables ?

**Response**. The article has two main objectives: (a) the comparison between Ethiopian and German cancer patients concerning mean scores of anxiety and depression, and (b) the analysis of the internal structure of the Ethiopian data set. It would be too far-reaching to analyze topics such as dependency of anxiety and depression on clinical variables also in the German sample. Such analyses have already done and published elsewhere. In reference Hinz, Herzberg et al. (2019) such analyses have been performed. Details of the German sample can also been found in that publication. We included information on cancer types and stages in the German comparison sample; however, analyses of the associations between these clinical variables and the HADS variables within the German sample would be beyond the scope of this article.

**52 Cancer incidence and mortality rates are lower* in low-income countries (LICs) than in HICs.**

*not for the individual cancer patient

**Response**. Cancer incidence and mortality are epidemiological concepts which refer to groups of people and not to individual people. The lower rates can be attributed to lower life expectancy in LICs, but when we use the concepts so as they are defined, the incidence and mortality coefficients are really lower in LICs.

**70 HADS have been conducted in high-income countries (HICs). A particular problem in LICs is**

**71 illiteracy.**

The question whether illiteracy has an influence on HADS results is very interesting and important.

It should be analysed further in detail.

How is the distribution of age, tumor stage, current treatment situation, time from diagnosis, chemotherapy/operation/radiation therapy/no treatment etc. between the patients with and without illiteracy ?

**Response**.

Illiteracy is really a methodological problem. Therefore, we already included Table 5 (now Table 6) to show the main differences between illiterate and literate patients, though this was not the main focus of the manuscript. The manuscript already comprises 7 tables. Adding a further table with sociodemographic and clinical differences between the illiterate and literate patients would enlarge the manuscript too much. However, we tested associations between illiteracy and several sociodemographic and clinical variables and included the results in the manuscript.

**114 German cancer patients**

**115 A group of German cancer patients served as controls. The patients were recruited in five**

**116 study centers in Germany. Further details of the sample are published elsewhere [30]. The**

**117 ethics committees from the five participating study centers approved the study. Data on**

**118 anxiety and depression in this sample has already been published [27].**

The references listed do not fit completely. In “reference 30” there are no HADS results published.

In “reference 27” is apparently no connection to the german cancer patients.

**464 27. Jimenez-Fonseca P, Calderón C, Hernández R, Ramón Y Cajal T, Mut M, Ramchandani**

**465 A, et al. Factors associated with anxiety and depression in cancer patients prior to**

**466 initiating adjuvant therapy. Clin Translat Oncol. 2018;20(11):1408–15.**

**467 doi: 10.1007/s12094-018-1873-9.**

**475 30. Mehnert A, Brähler E, Faller H, Härter M, Keller M, Schulz H, et al. Four-week**

**476 prevalence of mental disorders in patients with cancer across major tumor entities. J Clin**

**477 Oncol. 2014;32(31):3540–6. doi: 10.1200/JCO.2014.56.0086**

The data of the german patients should be included in the publication and described precisely.

Otherwise, no comparison with the ethiopean patients can be performed.

**Response**.

Reference [30] (Mehnert et al.) is correct. This article describes characteristics of the sample of the German cancer patients, though it does not present HADS scores.

However, reference [27] was not correct. We apologize for the mistake and replace it with the correct reference [28]. Thank you for this remark.

We now included two characteristics of the German sample: the frequencies of the most prevalent cancer diagnoses and the tumor stages. Since the focus is this article is on the Ethiopian cancer patients, we did not want to go into further detail with the German patients who only served as controls in this context.

**119 of 1,821 males and 1,964 females with a mean age of 58.3 119 years. Because of the differences in**

**120 the age and gender distribution between the Ethiopian and the German samples, we selected a**

**121 subsample of the German cancer patients matched to the Ethiopian one in those aspects. The**

**122 resulting German subsample consisted of 1,664 cancer patients, of which 638 (38.3 %) and**

**123 1,026 (61.7 %) were males and females, respectively. The mean age of this group was 48.0**

**124 years.**

It seems that the matching was only performed by looking at age and gender distribution.

What about tumor stage, current treatment situation, time from diagnosis, chemotherapy/operation/radiation therapy/no treatment etc. ?

**Response**. See response given to the problem of “matching” on page 1.

**188 high in the depression scale. All but one of the 14 items showed**

**189 higher means in the Ethiopian sample. The greatest difference between the Ethiopian and the**

**190 German cancer patients was found for the depression item D14, whereby an effect size of**

**191 greater than 1 was observed.**

It should be mentioned which item is higher in german patients (A3!)

**Response**. In the text we included the remark that the mean score of item A3 was slightly higher in the German sample.

**192 Concerning the three categories (no, doubtful, severe cases of anxiety and depression)**

**193 the results among the Ethiopian sample were as follows: *no* anxiety: *n*=129 (50.4 %), *doubtful***

**194 anxiety: *n* = 48 (18.7 %), *severe* anxiety: *n* = 79 (30.9 %); and *no* depression: *n* = 85 (33.2 %),**

**195 *doubtful* depression: *n* = 61 (23.8 %), *severe* depression: *n* = 110 (43.0 %). The corresponding**

**196 scores for the German sample were: *no* anxiety: *n*=995 (59.8 %), *doubtful* anxiety: *n* = 337**

**197 (20.3 %), severe anxiety: *n* = 332 (20.0 %); and *no* depression: *n* = 1172 (70.4 %), *doubtful***

**198 depression: *n* = 268 (16.1 %), *severe* depression: *n* = 224 (13.5 %).**

This description is very difficult to overview. It would be much more clear to use e.g. “no anxiety” 50,4% versus 59,8% in german versus ethiopean patients and a column chart.

In addition, a statistical analysis would be possible using correct matched pairs.

**Response**. According to the suggestion we arranged the percentages and case numbers in a new table. We had already performed statistical tests for the comparison between the Ethiopian and the German sample on the basis of mean scores. Therefore, we do not think that it is necessary to perform statistical tests for the percentages given in the new table.

**Reviewer #2:**

This is an important study that will make useful contributions to existing knowledge in related fields, especially with regard to Ethiopia. Given the cross-cultural comparisons, it might have been more appropriate to have submitted this to the Journal of Cross-Cultural Psychology for publication. However in all other respects it appears to be sound and useful. The manuscript is clearly written and logically coherent, the alphas of the related scales are sufficient, and the measures used are appropriate and valid, as well as the added check of comparison across cultural contexts. One can imagine that the disparate sizes of the Ethiopian and German samples made comparisons more difficult because the threshold for achieving significance would be different in the two samples, but this is not prohibitive. The final discussion section should acknowledge this limitation to the study however.

**Response.**

The discrepancy of the sample sizes has no effect on the estimates of mean scores, standard deviations, and coefficients of reliability. The only difference is that the larger sample size of the German sample gives more precise estimates of these coefficients. The effect sizes for the comparison between the Ethiopian and the German sample mainly depend on the mean score differences and the standard deviations; the samples sizes have a marginal impact on these coefficients.

Problems would result if we had analyzed factors within the German sample and within the Ethiopian sample (e. g., the dependency of anxiety and depression on the tumor stage). The larger sample size in Germany would result in statistically more significant effects than in the Ethiopian sample due to the greater n, even if the effects were equal in both samples. However, in the manuscript we do not report such comparisons.

If we had reduced the sample size of the German sample in order to approach the lower n in Ethiopia, the only effect would be that the estimates for the German sample would lose in precision.

Nevertheless, we acknowledged the effects of the disparate sample sizes and the imperfect matching of the samples in the Limitations section.

A few very minor specific recommended corrections...

35 - was/were. 'Data' is plural, so should be 'Data were...'.

**Response**. We corrected the word accordingly.

50 - Add 'Germany' to keywords.

**Response**. We added Germany in the keywords
