# Supplementary material for: The Hospital Anxiety and Depression Scale (HADS) applied to Ethiopian cancer patients
Source: PLoS One. 2020 Dec 3;15(12):e0243357. doi: 10.1371/journal.pone.0243357 (PMC7714130; doi:10.1371/journal.pone.0243357)
Supplement: Attachment — Submitted filename: Response to Reviewers R2.docx [file pone.0243357.s003.docx]

**Response to Reviewers**

Reviewer #1:

Please include the number of the german sample in the text and mention that the tumor stage was different in the Ethiopian versus the german sample (Stage I 7,8% versus 22,2%) which can influence the HADS results.

Response.

We already gave the number of the German sample in the methods section.

In the Limitations section we now repeated the number and acknowledged that the differences in the distributions of the tumor stages limit the comparability of the two samples.

Reviewer #2 had no additional remarks.
